# How transport coverage shapes access to opportunities: a national-scale isochrone analysis of bus network accessibility in England

**DOI:** 10.3389/fdata.2026.1942887

**Published:** 2026-10-22

**Authors:** Sam Townley, Federico Botta

**Affiliations:** Department of Computer Science, University of Exeter, Exeter, United Kingdom

**Keywords:** bus networks, human mobility, isochrones, public transport, rural–urban divide, social exclusion, spatial inequality, transport accessibility

## Abstract

Equitable access to opportunities is a cornerstone of social inclusion, and public transport plays a central role in delivering it for the millions of households without access to a private car. Yet most accessibility research has focused on case studies of individual cities, while transport funding decisions in the UK are typically made at regional or national scales. This paper evaluates the infrastructure-based accessibility of bus networks in England, together with their connectivity to key destinations such as places of employment, healthcare and education. We propose a scalable, isochrone-based methodology that compares the area reachable by bus to the area reachable by car within a fixed travel-time budget, and combines it with a destination-count ratio that quantifies the share of destinations reachable by bus relative to those reachable by car. Applied across all 33,755 Lower-layer Super Output Areas (LSOAs) in England, the analysis reveals deep and spatially structured inequalities in mobility-mediated access to opportunities. We show that 61.4% of LSOAs, home to 60.9% of the resident population, reach less than 1% of the equivalent car-accessible area by bus, and that 71% of LSOAs (70.6% of the resident population) reach fewer than 1 in 20 of the destinations a car user could reach. Spatial autocorrelation analysis reveals widespread clustering of low accessibility outside major cities, while urban areas show finer-grained, heterogeneous patterns of disadvantage. The findings highlight rural-urban inequalities and within-city heterogeneity, and point to areas where targeted bus-network investment could yield significant gains in social inclusion. The methodology relies only on openly available timetable and street-network data and is therefore transferable to other countries and applicable at finer geographical scales.

## 1 Introduction

Affordable and reliable public transport is central to providing uniform access to opportunities (Gates et al., 2019; Lucas et al., 2012), particularly for the 22% of households in the UK that do not own a car (Department for Transport, 2025). An estimated 1.5 million people in the UK are at high risk of being transport poor, often living in low-income neighborhoods with limited infrastructure, and vulnerable populations in rural areas are particularly exposed to these effects, with transport-related deprivation having a stronger negative impact on personal wellbeing than in urban settings (Gates et al., 2019). Bus services carry much of this burden, as they are the only public-transport mode with near-universal national coverage. In the UK, transport planning and investment is coordinated by the Department for Transport, while service operation is largely devolved to private companies that must run routes for profit. This is particularly challenging for buses, which often require subsidies: in 2019–2020, over 40% of bus revenue in Great Britain came from government grants (Preston, 2023). Despite this level of subsidy, the accessibility a bus network delivers depends on far more than fare cost: connectivity, travel time and the range of destinations served all shape a network's social value and travelers' mode choices (Gates et al., 2019). Most empirical accessibility research has been built around case studies of large cities such as London or Helsinki (Ford et al., 2015; Fink et al., 2024), in part because the spatial scale of analysis has historically been constrained by the speed of routing algorithms. As a result, both the national-scale distribution of bus accessibility and its relationship with other measures of area-level disadvantage remain incompletely characterized. Improving the range of destinations reachable by public transport has been shown to increase incentives to travel and to improve the quality of life of those who depend on it (Niedzielski and Boschmann, 2014), and bus routes that fail to prioritize key services can lower individuals' chances of employment (Gates et al., 2019), but these effects have rarely been examined outside individual cities. Modern routing engines such as *R*^5^ and its Python interface r5py are one to two orders of magnitude faster than earlier tools (Higgins et al., 2022), making national-scale, fine-grained analyses tractable for the first time and creating an opportunity to move beyond mapping where accessibility disparities occur to identifying who experiences them and how they relate to conventional deprivation indicators. In this paper, we use *accessibility* in its transport-geography sense: the ease with which destinations or activities can be reached using a given transport system (Geurs and van Wee, 2004; Dalvi and Martin, 1976). Standard accessibility measures combine four dimensions: land-use, transportation, temporal, and individual (Geurs and van Wee, 2004). Here, we primarily target the first two. To quantify accessibility we use *isochrones*, contours of equal travel time from an origin point (Galton, 1881). Free-flow isochrones based on uniform travel speeds are known to overestimate accessibility substantially (Śleszyński et al., 2023; van den Berg et al., 2018); we therefore use a routed driving isochrone as a baseline against which a routed bus isochrone is compared, taking the car as a meaningful upper bound on local reach within a given time budget (Gates et al., 2019). Note that we treat driving as a theoretical maximum reach afforded by the road network under no-traffic, free-flow conditions. Following (Śleszyński et al., 2023; Gates et al., 2019), we work with *access areas* (destinations reachable from an origin) rather than service areas, as the access framing aligns with operational definitions of social exclusion. Our contribution is threefold. First, we introduce a scalable, isochrone-based methodology for jointly assessing infrastructural and destination-based accessibility at national scale, using only openly available timetable and street-network data. Second, we apply this methodology to England's bus network at the level of all 33,755 Lower-layer Super Output Areas (LSOAs), fitting a multivariate spatial regression on the resulting indicators to identify the socioeconomic covariates that predict bus-mediated accessibility. Third, we show that our destination-based accessibility indicator is only weakly correlated with the English Index of Multiple Deprivation, providing evidence at national scale that mobility-mediated accessibility captures a dimension of area-level disadvantage that conventional deprivation indices do not fully reflect. By framing accessibility as a function of human mobility patterns rather than infrastructure alone, the paper directly addresses how mobility shapes access to opportunities at national scale, and how the resulting spatial patterns of bus-mediated accessibility relate to conventional indicators of area-level disadvantage.

## 2 Materials and methods

### 2.1 Data

The geographic unit of analysis is the Lower-layer Super Output Area (LSOA), the second-smallest subdivision used in UK census statistics, with each LSOA covering 400–1,200 households (roughly 1,000–3,000 residents). England contains 33,755 LSOAs (Office for National Statistics, 2022c); population-weighted centroids (Office for National Statistics, 2025b) are used as origin points, and Middle-layer Super Output Areas (MSOAs) (Office for National Statistics, 2024b) are used solely to batch the routing computations. LSOA, MSOA, region (Office for National Statistics, 2025c) and 2024 county/unitary-authority boundaries (Office for National Statistics, 2025a) are taken from the Office for National Statistics (ONS) Open Geography Portal (Office for National Statistics, 2024a). Street-network data come from OpenStreetMap (England extract, GeoFabrik Geofabrik, 2025, snapshot dated 19 January 2026), and bus timetables from the Bus Open Data Service (BODS) (Department for Transport, 2020) in General Transit Feed Specification (GTFS) format. Since 2020, all English bus operators have been legally required to publish to BODS, ensuring near-complete coverage. For destinations, we use the 2019 Department for Transport Journey Time Statistics (Department for Transport, 2015). The 2019 edition provides locations of schools, GPs and hospitals, and assigns each LSOA an employment-center size (small: < 500 jobs; medium: 500–4,999; large: ≥5, 000). Settlement type is taken from the ONS six-class Rural–Urban Classification (Office for National Statistics, 2025d). Small-area deprivation is measured using the 2025 English Index of Multiple Deprivation (IMD) (Ministry of Housing, Communities and Local Government, 2025), published at LSOA level by the Ministry of Housing, Communities and Local Government. The household composition variables total population (table TS001) and the share of households without access to a car or van (table TS045) are taken from the 2021 UK Census via ONS Nomis (Office for National Statistics, 2022a,b).

### 2.2 Routing engine and isochrone computation

Routing is performed using r5py (Fink et al., 2022), a Python wrapper around Conveyal's *R*^5^ engine (Conveyal, 2018; Conway et al., 2017), which has been adopted in several recent accessibility studies (Fink et al., 2024; Chen and Botta, 2025, 2026; Lopes et al., 2025) for its ability to compute multi-modal travel-time matrices one to two orders of magnitude faster than earlier engines (Higgins et al., 2022; Bast et al., 2016). We use r5py, Geopandas and Shapely (Fink et al., 2022; den Bossche et al., 2024; Gillies et al., 2025) to reconstruct each isochrone as a 2D concave-hull polygon (with a 20 m buffer) over a grid of destinations whose travel times fall within the time budget. We use a 40-minute travel-time budget, motivated by the 2023 UK statistic of a 37-minute average bus commute (Department for Transport, 2024). Weekday peak commuting is modeled by a Monday 26 January 2026, 08:30 departure with a 60-minute window (Fink et al., 2024); the same window is applied to both modes, on the assumption that traffic affects buses and cars comparably (Benenson et al., 2010), though we note this assumption does not necessarily always hold. We also highlight that R5 does not model road congestion or traffic, so car-isochrones are unaffected by the choice of departure time. To keep the analysis tractable at national scale, for each MSOA we crop the OSM street network to the bounding box that extends 70 km beyond every LSOA population-weighted centroid within that MSOA, and batch-compute isochrones for all LSOAs in that MSOA using the same cropped network. Every LSOA in the analysis therefore has at least 70 km of routed street network available in every direction from its origin. For the isochrone calculation in r5py, the travel-time grid is set to 250 m for bus journeys and 500 m for driving; we use the 25th-percentile travel time across the departure window for buses (modeling the natural tendency of regular travelers to leave the house close to a scheduled departure) and the default 50th-percentile for cars. Multi-line outputs are unioned into polygons; an example is shown in Figure 1. Note that for bus traveling we also allow the option of walking, as this may be needed to walk to the closest bus stop, or between stops if taking multiple buses in one journey. LSOAs producing an empty or single-point geometry, or physically implausible isochrones where traveling by bus is significantly faster than by car (*R*_*A*_ > 1), are excluded: in total, 97 LSOAs (~0.3%) are removed across both modes. Sensitivity analyses across the bounding-box size, departure time, GTFS catchment radius and travel-time percentile produced no systematic change in these failures, suggesting they reflect routing artifacts rather than parameter choices; we discuss potential mitigations in Section 4. In order to ensure that our results hold across a range of parameter choices, we re-ran the full analysis on a stratified 483-LSOA subsample under five alternative parameter settings that perturb one dimension of the specification at a time: a 20-minute and a 60-minute isochrone; an off-peak (Monday 14:00) and a weekend (Saturday 10:00) departure time; a matched bus and car travel-time percentile; and a matched 250 m isochrone grid for both modes. Results are reported in Supplementary Table 8: absolute levels scale with the time budget as expected, the percentile choice reduces bus reach by around 25–35%, and the departure time and grid resolution move the metrics by less than five percent. In every scenario the rank ordering of LSOAs on *R*_*A*_ and *R*_*D*_ is preserved (Spearman ρ≥0.76 for *R*_*A*_ and ρ≥0.44 for *R*_*D*_, with the lowest *R*_*D*_ correlation reflecting a floor effect at the shortest budget where many LSOAs reach zero destinations).

**Figure 1:**
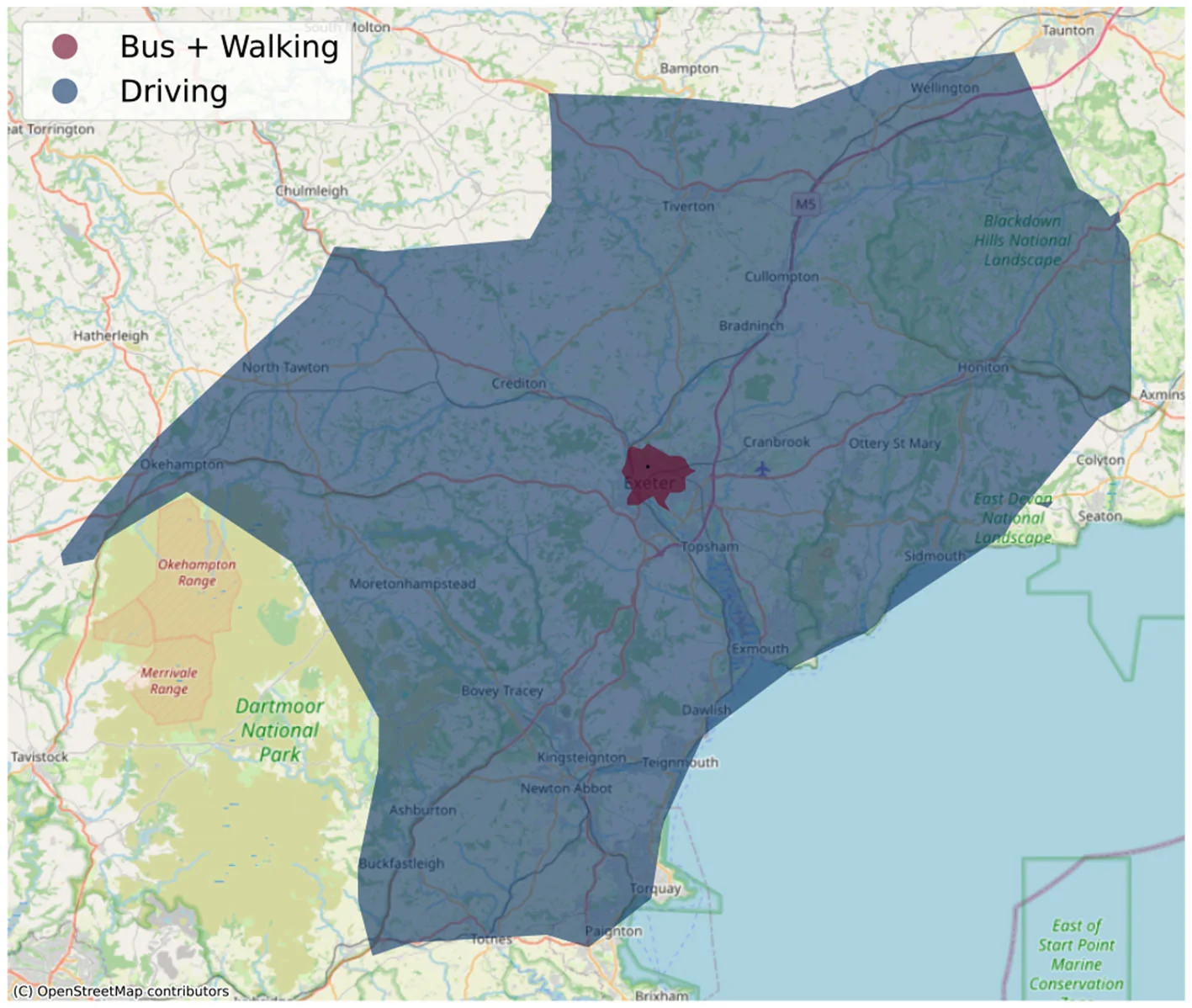
Isochrone calculation. For each starting point in our analysis, we use r5py to calculate bus and car 40-minute isochrones. Here, we show an example for LSOA E01034628, located on the University of Exeter campus. We can easily see that the car isochrone results in a significantly larger accessible area compared to the bus isochrone. © OpenStreetMap contributors.

### 2.3 Indicators of accessibility

We focus on indicators that align with the UK government's Leveling Up Fund evaluation criteria, in particular “time taken to/from the nearest large employment center and/or local services such as health centers and schools” (Department for Transport, 2022). By using metrics that map onto investment criteria, the resulting indicators can be readily linked to policy decisions.

#### 2.3.1 Isochrone area ratio

Earlier studies have defined the “degree of accessibility” as the ratio of inhabitants in a real service area to those in an idealized service area (Śleszyński et al., 2023). Reliable population data at high spatial granularity is, however, difficult to obtain at national scale. We therefore adopt the alternative formulation, also proposed in the same literature, that compares the two areas directly:


$$R_{A}=\frac{A_{b}}{A_{c}},$$

where *A*_*b*_ is the area of the bus isochrone and *A*_*c*_ that of the car isochrone, both computed in square kilometers after re-projecting the polygons to a cylindrical equal-area projection (PROJ string +proj=cea). With the car isochrone treated as a notional upper bound on local reach, *R*_*A*_∈[0, 1] measures the ratio of bus-reachable area to car-reachable area within the same time budget. Low values indicate scope for improving infrastructure, while high values indicate well-connected travel regions.

#### 2.3.2 Destination-count ratio

Area-based indicators alone can be misleading, since they do not distinguish between coverage of populated activity centers and coverage of empty space. We therefore complement *R*_*A*_ with a *destination-count ratio*, defined as


$$R_{D}=\frac{N_{b}}{N_{c}},$$

where *N*_*b*_ and *N*_*c*_ are the numbers of destination points reachable within 40 minutes by bus and by car, respectively, obtained by spatially joining the destination dataset to each isochrone. Destinations are drawn from the eight types provided by the 2019 Journey Time Statistics dataset: small, medium and large employment centers; primary, secondary and further-education establishments; general practitioners and hospitals. Each destination point contributes equally to the ratio. We also report the three category-specific ratios (employment, education and healthcare) constructed by restricting *N*_*b*_ and *N*_*c*_ to the corresponding subset of destination types. Because JTS 2019 supplies employment centers in three ordinal size bands rather than continuous job counts, and does not report per-establishment capacity for schools, GPs or hospitals, *R*_*D*_ measures the coverage of destination *points* rather than the volume of jobs, pupils or patient capacity accessible from each origin. To assess whether the count-based construction materially affects the results, we compared it against four alternative weightings of the employment component in which each employment center is weighted by its band's assumed job count rather than counted equally: the JTS band lower bounds (100/500/5,000), the band midpoints (300/2,750/7,500), an upper-heavy variant that gives greater weight to large centers (300/2,750/15,000), and the geometric mean of the band bounds (224/1,580/8,660). The four schemes span a range from 25:1 to 50:1 in the ratio of the large-band to the small-band weight, against a ratio of 1:1 under the count baseline. The resulting weighted employment ratios correlate with the count-based version at Spearman ρ≥0.93 and shift the share of LSOAs below the headline threshold by less than one percentage point in every case (Supplementary Table 6). For LSOAs where neither isochrone contains any destinations, we set *R*_*D*_ = 0, since accessibility in such cases is in any event very poor. An example for a central-Exeter LSOA is given in Figure 2. Combinations of the two indicators are diagnostic of different network characteristics: regions with low *R*_*D*_ but relatively high *R*_*A*_ are good candidates for re-routing existing services to better connect already-covered areas to key destinations; regions with low *R*_*A*_ but relatively high *R*_*D*_ suggest that bus routes are designed to prioritize access to destinations rather than general travel distance; and regions with low values on both indicators may instead need new or better designed routes.

**Figure 2:**
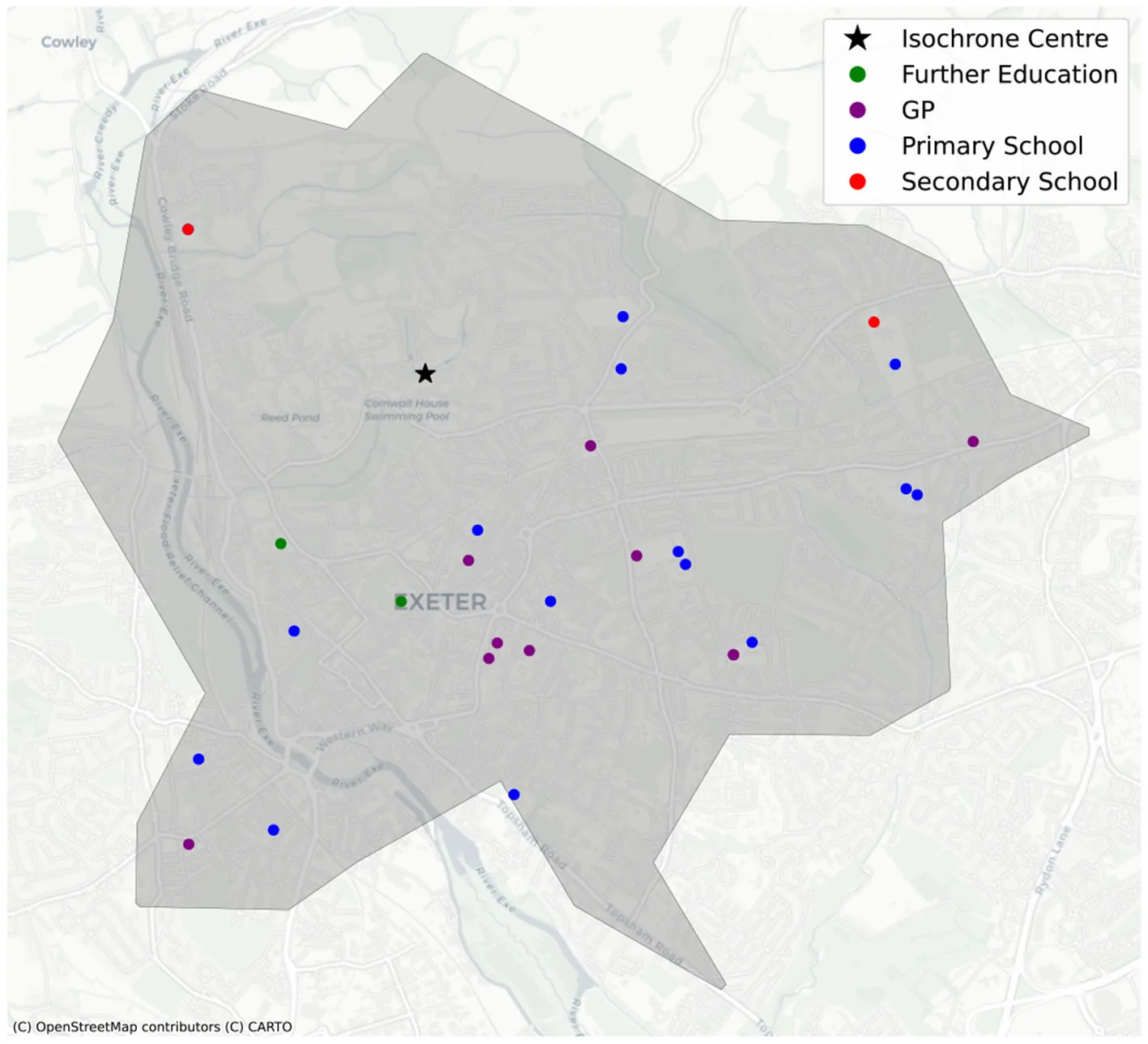
Accessibility of destination. For each isochrone, we retrieve the number of destinations, across a number of categories, that are contained in it. This allows us to study how the design of the bus network shapes the type and number of destinations accessible and compare this to our baseline of driving. Here, we show an example isochrones from LSOA E01034628 (University of Exeter campus) and the destinations contained in it. © OpenStreetMap contributors.

#### 2.3.3 Spatial autocorrelation

To examine the spatial structure of *R*_*A*_ and *R*_*D*_, we compute the global Moran's *I* and the corresponding Local Indicators of Spatial Association (LISA) (Tobler, 1970; Zhou and Lin, 2008; Anselin, 1995) on a queen-contiguity weights matrix over LSOAs, using the *libpysal* and *esda* packages from PySAL (Rey and Anselin, 2007). The Isles of Scilly (LSOA ID: E01019077) are excluded from the weights matrix because of their geographical isolation. Statistical significance is assessed via 999 random permutations at *p* < 0.05, following comparable studies (Chen and Botta, 2025). Global Moran's *I* values, expected values under the null, *z*-scores and permutation-based *p*-values for each principal indicator are reported in Supplementary Table 9; all six indicators reject the null of no spatial dependence at the smallest attainable permutation *p*-value. With more than 33, 000 local tests, a nominal α = 0.05 threshold will produce a non-trivial number of false-positive LISA classifications by chance; our substantive interpretation of the LISA maps therefore rests on the aggregate spatial patterns visible in the maps rather than on any single LSOA's cluster classification. Where Pearson correlation coefficients are reported alongside the maps and scatter plots in Section 3, we treat them as descriptive summaries of joint distributions; with *N*≈33, 000 and strong spatial dependence, the accompanying parametric *p*-values understate the true uncertainty, and substantive inference is instead carried by the spatial regression models in Section 3.5.1.

### 2.4 Regression on socioeconomic covariates

To identify covariates of the destination-count ratio *R*_*D*_, we fit an ordinary least squares (OLS) regression of log(*R*_*D*_) on a rural indicator (RUC21 code starting with R), the LSOA-level score of the 2025 English Index of Multiple Deprivation (IMD) (Ministry of Housing, Communities and Local Government, 2025), the share of households without access to a car or van (Census 2021 table TS045 Office for National Statistics, 2022b), and population density derived from the Census 2021 population (table TS001 Office for National Statistics, 2022a) and the LSOA polygon area. Continuous predictors are z-standardized so that coefficients can be compared on a common scale, and we include a multiplicative interaction between IMD and the rural indicator to allow the deprivation gradient of *R*_*D*_ to differ between settlement types. Standard errors on the OLS estimates are heteroskedasticity-robust (HC3) (MacKinnon and White, 1985). The 97 LSOAs flagged as invalid in Section 2.2 and the geographically isolated Isles of Scilly (E01019077) are excluded, leaving *N* = 33, 657 LSOAs in the estimation sample. For the small fraction of LSOAs with *R*_*D*_ = 0, a numerical offset of ε = 10^−4^ is added prior to log-transformation so that these observations remain in the estimation sample; the offset is roughly an order of magnitude below the smallest non-zero *R*_*D*_ and does not affect the qualitative results. Because the residuals of the OLS fit exhibit strong spatial autocorrelation (global Moran's *I* = 0.66, permutation *p* < 0.001; see Section 2.3.3), we complement OLS with three spatial specifications: a spatial error model (SEM), in which the residuals of adjacent LSOAs are correlated through an unobserved autoregressive process with parameter λ; a spatial lag model (SLM), in which the outcome log(*R*_*D*_) itself depends on the average of its neighbors' values through parameter ρ; and a combined spatial-autoregressive-with-error (SARAR) model (Kelejian and Prucha, 2010),


$$\begin{array}{l} \log\left(R_{D}\right)=\rho W\log\left(R_{D}\right)+X\beta +u,\;u=\lambda Wu+\epsilon , \end{array}$$ (1)

which combines both. In the spatial specifications that include a spatial lag of the outcome (SLM and SARAR), reported coefficients correspond to the *direct* marginal effect of a covariate on the outcome; the associated *total* impact, which additionally captures the spillover of that change through the lagged outcome, is approximately β/(1−ρ) (LeSage and Pace, 2009). The choice of specification is guided by the standard battery of Lagrange-multiplier (LM) diagnostics on the OLS residuals (Anselin, 1988; Elhorst, 2010). As reported in Table 1, both the classical and the robust LM statistics reject the null of no spatial dependence in both the residual structure and the outcome (all *p* < 0.001), consistent with the presence of both types of dependence simultaneously. We accordingly take SARAR as the preferred specification and report OLS, SEM and SLM as robustness comparators in Table 2. All spatial models are fitted with the generalized method-of-moments estimators implemented in the PySAL (Rey and Anselin, 2007) spreg module (GM_Error, GM_Lag, and GM_Combo_Het), which have well-established properties at the sample size considered here (Arraíz et al., 2010).

**Table 1:** Lagrange-multiplier (LM) diagnostics for spatial dependence in the OLS regression of log(*R*_*D*_) on rural, IMD, car-less-household share, population density, and the IMD × rural interaction.

Test	Statistic	df	*p*
LM-Error	40,714.59	1	< 0.001
LM-Lag	37,210.44	1	< 0.001
Robust LM-Error	4,446.03	1	< 0.001
Robust LM-Lag	941.88	1	< 0.001
SARMA (joint)	41,656.47	2	< 0.001

Tests follow Anselin (1988) and Elhorst (2010). LM-Error and LM-Lag test the null of no spatial dependence in the residuals and in the outcome, respectively; the robust versions test each in the presence of the other; SARMA is a joint test for either type of dependence. Weights are row-standardized queen contiguity over LSOAs. All statistics are chi-squared distributed with the indicated degrees of freedom. *N* = 33,657.

**Table 2:** Four-way comparison of OLS, spatial error (SEM), spatial lag (SLM) and combined spatial lag + error (SARAR) estimates for the regression of log(*R*_*D*_+ε).

	OLS		SEM		SLM		SARAR	
Predictor	β	SE	β	SE	β	SE	β	SE
Intercept	-3.498	(+0.005)	-3.457	(+0.013)	-1.732	(+0.038)	-1.965	(+0.047)
Rural	-0.965	(+0.016)	-0.929	(+0.018)	-0.557	(+0.014)	-0.692	(+0.026)
IMD (z, urban)	-0.163	(+0.007)	-0.186	(+0.007)	-0.093	(+0.005)	-0.134	(+0.005)
Car-less household share (z)	+0.656	(+0.007)	+0.450	(+0.009)	+0.349	(+0.008)	+0.380	(+0.009)
Population density (z)	+0.022	(+0.005)	+0.007	(+0.003)	+0.003	(+0.004)	+0.001	(+0.002)
IMD × Rural	+0.278	(+0.023)	-0.006	(+0.017)	+0.162	(+0.016)	+0.064	(+0.024)
λ (spatial error)	—	—	+0.771	(+0.017)	—	—	+0.540	(+0.014)
ρ (spatial lag of *y*)	—	—	—	—	+0.496	(+0.011)	+0.424	(+0.013)

Continuous predictors are z-standardized. Spatial models use row-standardized queen-contiguity weights; the Isles of Scilly are excluded. LM diagnostics on the OLS residuals (see main text) motivate the SARAR specification. *N* = 33,657.

Spatial neighborhoods are defined by a row-standardized queen-contiguity weights matrix *W* over the estimation sample, matching the specification used for the spatial-autocorrelation analysis in Section 2.3.3. Because the choice of *W* can influence the estimated autoregressive parameters and, less commonly, the covariate coefficients, we test whether our SARAR estimates are stable across alternative definitions. In addition to queen contiguity, we refit the SARAR specification with *k* = 4 and *k* = 8 nearest-neighbor weights constructed from LSOA population-weighted centroids and row-standardized. Results are reported in Supplementary Table 4: covariate signs, statistical significance and relative orderings are preserved across all three weights specifications, and the spatial-lag parameter ρ is essentially invariant to the choice of *W*. The spatial-error parameter λ increases with the density of the neighborhood definition, from λ = 0.54 under queen to λ = 0.80 under KNN-8, so we interpret λ throughout as an indicator of the presence rather than the precise magnitude of unmeasured spatially clustered factors. Because *R*_*D*_ is a ratio of bus-reachable to car-reachable destinations, a positive coefficient in the log*R*_*D*_ regression can in principle reflect either an increase in the number of bus-reachable destinations (numerator) or a decrease in the number of car-reachable destinations (denominator), or a combination of the two. To distinguish these mechanisms, we refit the SEM specification with log(total bus destinations+1) and log(total car destinations+1) as separate outcomes (with the standard +1 offset conventional for log-count outcomes on non-negative integer data). Because log*R*_*D*_≈log(bus)−log(car), the coefficient on each predictor in the *R*_*D*_ regression is closely approximated by the difference of its coefficients in the two component regressions, and the decomposition therefore allows us to attribute the estimated *R*_*D*_ effects to their bus-side and car-side origins. The decomposition is reported in Table 3 and interpreted in Section 3.5.2. All regression analyses were carried out in Python 3.12 using the statsmodels (Seabold and Perktold, 2010) and PySAL (Rey and Anselin, 2007) libraries; the full analysis code and processed LSOA-level dataset are archived at (Townley, 2026).

**Table 3:** Decomposition of the SEM coefficients from Table 2 into their bus-side (numerator) and car-side (denominator) contributions.

	log(bus dest.)		log(car dest.)		log(*R*_*D*_)	
Predictor	β	SE	β	SE	β	SE
Rural	-0.983	(0.014)	-0.062	(0.009)	-0.929	(0.018)
IMD (z)	-0.220	(0.005)	-0.025	(0.004)	-0.186	(0.007)
Car-less household share (z)	+0.522	(0.007)	+0.041	(0.005)	+0.450	(0.009)
Population density (z)	+0.014	(0.003)	-0.000	(0.002)	+0.007	(0.003)
IMD x Rural	-0.038	(0.013)	-0.023	(0.008)	-0.006	(0.017)
λ (SEM error autocorr.)	+0.762	(0.013)	+0.904	(0.008)	+0.771	(0.017)

Each column reports SEM estimates from the same specification with a different outcome: log(total bus destinations+1), log(total car destinations+1), and $\log\left(R_{D}+10^{-4}\right)$. All coefficients are estimated on the same *N* = 33, 657 LSOAs with row-standardized queen-contiguity spatial weights. Because log*R*_*D*_≈log(*bus*)−log(*car*) up to the difference between the εand +1 offsets, the bus-side coefficient minus the car-side coefficient approximates (but does not exactly equal) the ratio coefficient. For LSOAs where the car destination count is zero, both offsets ensure the outcomes remain defined; the resulting approximation error is small in aggregate but the identity should not be read as exact. Standard errors in parentheses.

## 3 Results

### 3.1 Bus coverage

We start from the infrastructure-level perspective, comparing the geographic reach of bus and car isochrones. At the national scale, 61.4% of LSOAs reach less than 1% of the area accessible by car within 40 minutes when traveling by bus, and 97.8% of LSOAs reach less than 5%. Because LSOAs are designed to have roughly equal populations (400–1,200 households each), LSOA-share statistics are empirically very close to population-share statistics throughout the analysis: weighting by the Census 2021 LSOA population, the two figures above become 60.9% and 97.8% respectively, and the same near-equivalence holds for all headline shares reported below (differences below one percentage point in every case; see Supplementary Table 7 for the full comparison). These figures point to large inequalities in ease of movement for populations who cannot use private transport, often because of financial or health-related constraints. As Figure 3 shows, most of the inequality is driven by limited bus coverage. The bus-area distribution is strongly right-skewed, with a peak around 10km^2^, a median of 22.7km^2^ and a maximum of 233km^2^, well below typical car-isochrone areas. Car areas are by contrast more symmetrically distributed and have a much larger standard deviation, reinforcing the use of driving as a stable accessibility baseline; the small cluster of low values is likely to correspond to LSOAs in dense city centers, where low speed limits, frequent turns and one-way systems may reduce the reachable area. Mapping *R*_*A*_ across England (Figure 4a) shows that large parts of the country experience poor relative bus coverage, with hotspots concentrated in the largest cities. London is the most balanced large region in terms of area-equivalence between bus and car, and the only large significant cluster of relatively high *R*_*A*_ values lies in central London (Figure 4b). Other major cities have high values on average but no comparable cluster, indicating hyper-local rather than city-wide gains; Newcastle is illustrative, with a few high-value pockets surrounded by non-significant LISA values, plausibly reflecting the role of long-distance bus services and unmodelled metro systems. Outside the cities, we observe extensive and statistically significant clustering of low values, indicating that bus-mediated transport disadvantage is widespread rather than localized, and is the dominant pattern in rural settlements. Several factors plausibly contribute: rural bus isochrones tend to be smaller and elongated along population corridors, lower service frequency increases waiting times, and faster, straighter rural roads inflate car isochrones, depressing *R*_*A*_. Comparing the two modes by settlement type (Figure 5a) confirms that car-isochrone areas are ssimilarly heterogeneous in both rural and urban LSOAs, while bus-isochrone area is the clear differentiator; *R*_*A*_ correlates strongly with bus-isochrone area itself (Pearson *r* = 0.78, *p* < 0.001; Figure 5b).

**Figure 3:**
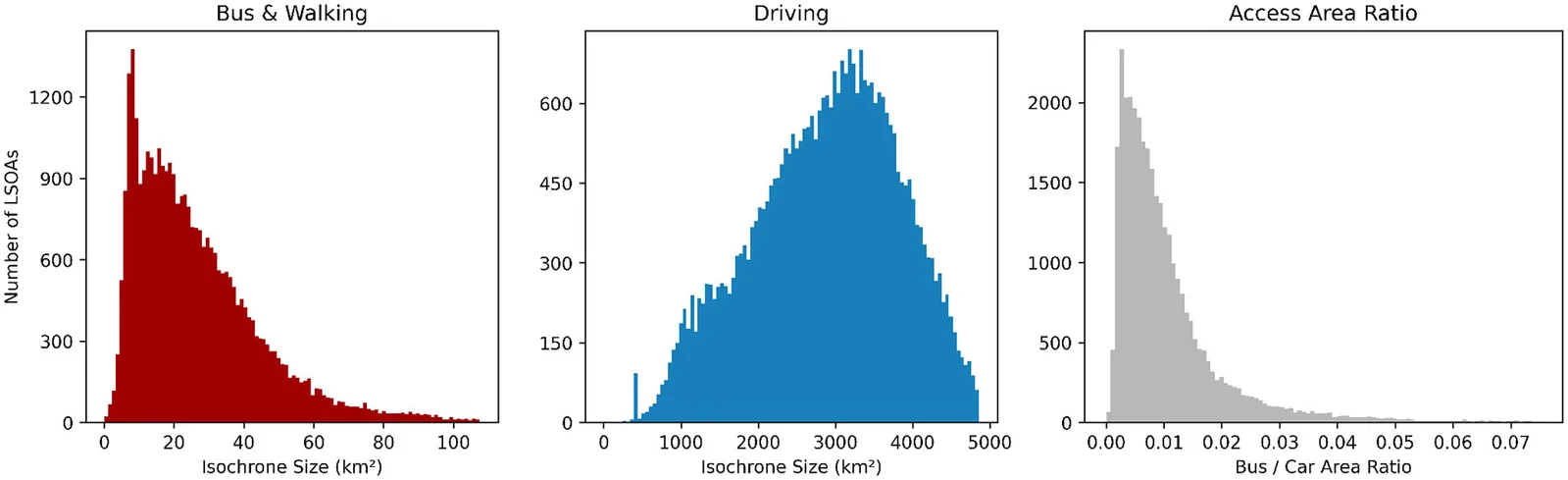
Accessibility distributions. For each LSOA, we calculate the area of 40-minute isochrones when traveling by bus or car. Here, we depict the distributions of the areas (up to the 95th percentile), as well as that of the bus-to-car area ratio *R*_*A*_. Visual inspection highlights a right-skewed distribution for bus accessibility, whereas driving accessibility shows a more symmetric distribution and with higher mean, resulting in the ratio being right-skewed and with most values close to zero.

**Figure 4:**
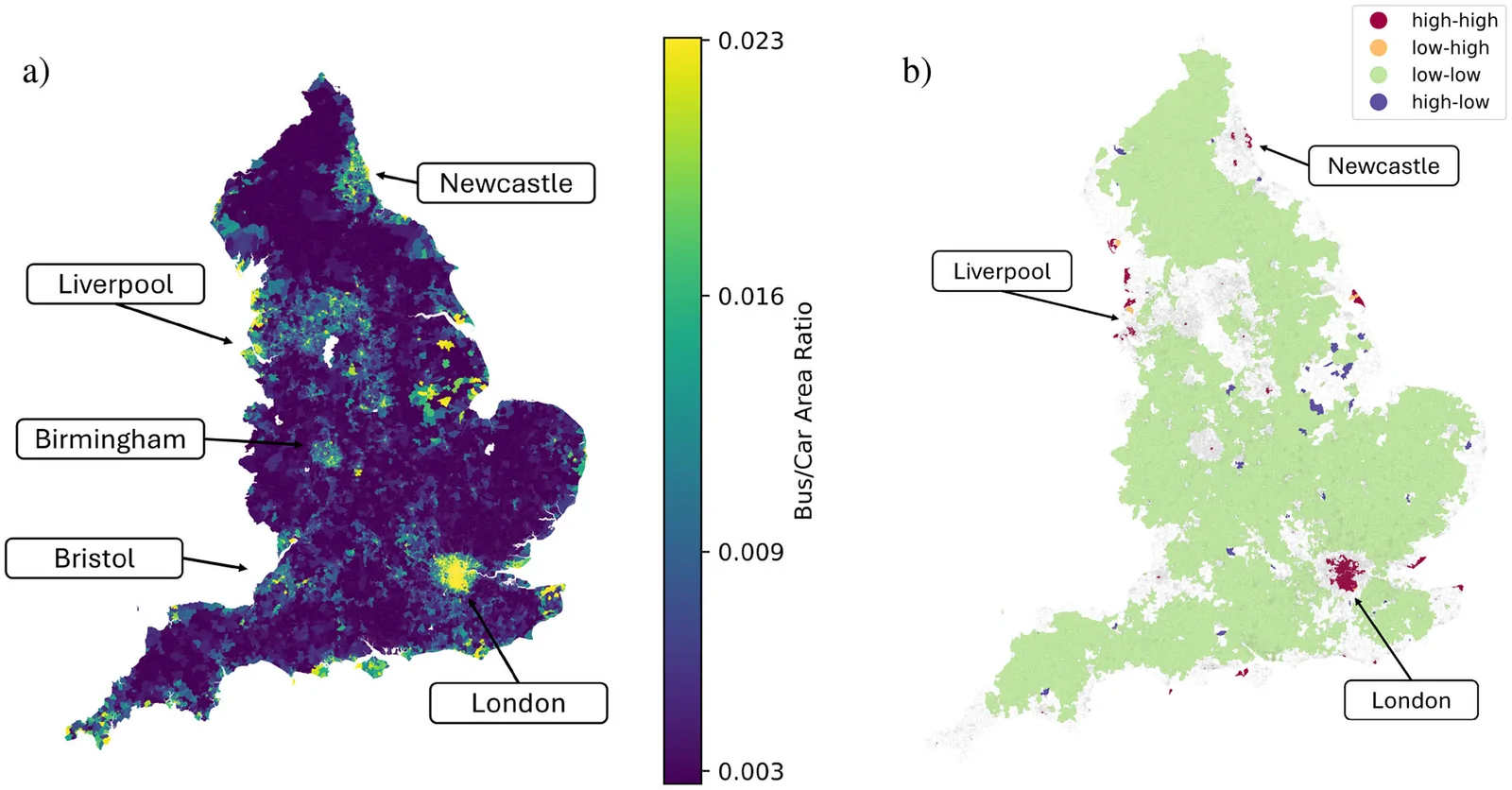
Accessibility maps. We analyse the difference in accessibility by bus and car via the ratio *R*_*A*_, which compares the area of a 40-minute bus isochrone to that of a driving isochrone. **(a)** We depict here a map of *R*_*A*_, highlighting some areas where the ratio achieves higher values. Visual inspection suggests that this happens in major metropolitan areas. **(b)** We use Local Indicators of Spatial Association to identify spatial clusters. London appears as the main cluster with high values; Liverpool and Newcastle have small areas of high value clusters, but do not achieve widespread significant clustering similar to London (values displayed are significant at *p* < 0.05). Note that the color scale runs from the 10th to the 90th percentile of *R*_*A*_. LSOAs with values outside this range are shown in the endpoint colors.

**Figure 5:**
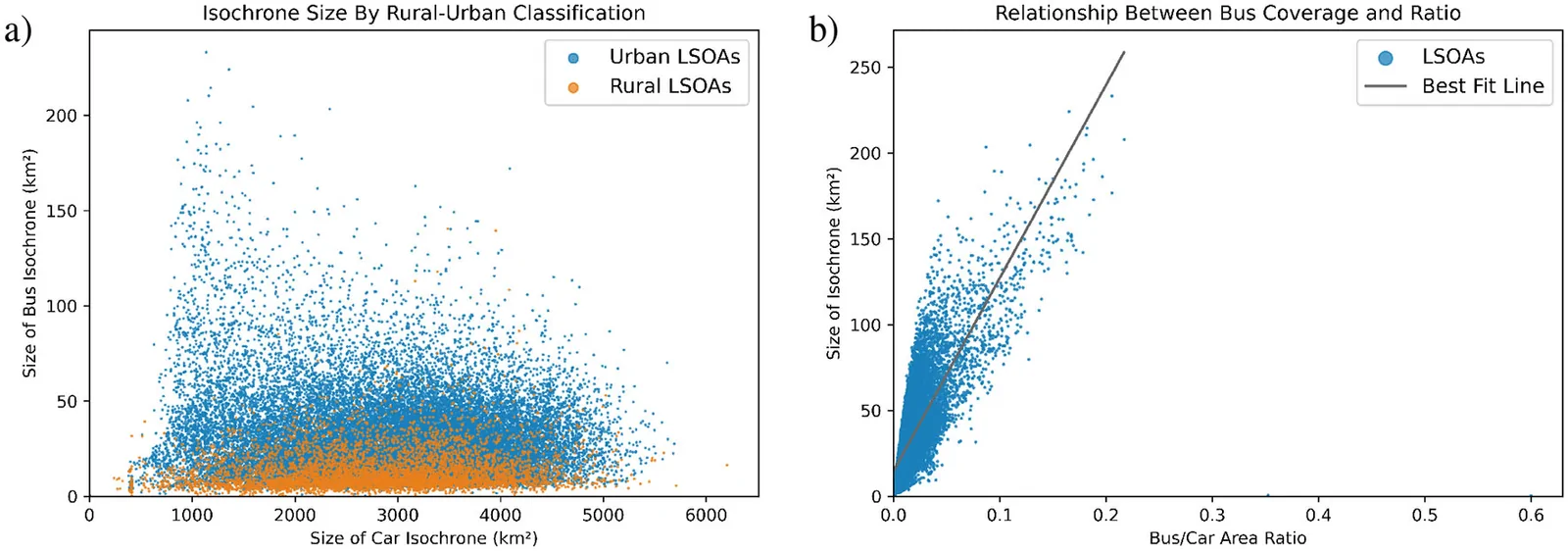
Urban and rural accessibility. To investigate whether there is an urban vs. rural divide, we study the relationship between the size of bus isochrones and car isochrones separately for urban and rural LSOAs. **(a)** We note that urban LSOAs tend to have a wider spread of areas for bus isochrones, whereas rural LSOAs tend to have small bus isochrones but can achieve comparatively large areas by car. **(b)** We observe that LSOAs with higher *R*_*A*_ values correspond to LSOAs with larger bus isochrones, suggesting that bus isochrones drive the variability in *R*_*A*_.

### 3.2 Destination accessibility

Lower geographic coverage matters for social inclusion only insofar as it translates into reduced access to actual destinations. In fact, one might argue that bus routes should prioritize access to destinations over general travel distance, particularly in situations where funding for route or frequency expansion is limited. We therefore examine *R*_*D*_, the bus-to-car destination-count ratio, across three categories: employment, healthcare and education. Their density (Figure 6a) shows clusters around major cities but a reasonable spread across the country; healthcare is the most clustered category, while employment and education are more uniformly distributed (though we note that the distribution of employment centers is driven by the design of the data set, which assigns one such center per LSOA). The overall destination-count ratio *R*_*D*_ is shown in Figure 6b. Compared to *R*_*A*_, hotspots are smaller and more numerous, and London occupies a less dominant share. The mean *R*_*D*_ across England is 0.048, four times larger than the mean *R*_*A*_ of 0.012: bus isochrones, although geographically smaller, are evidently shaped to maximize coverage of nearby destinations. Nevertheless, around 71% of LSOAs have *R*_*D*_ < 0.05 (70.6% of the resident population under population weighting), meaning that bus-reliant residents in those areas can reach at most one in twenty of the destinations a car user could reach. Because 83.5% of LSOAs are classified as urban, this figure must apply to substantial parts of the urban population as well, indicating that destination-level disadvantage is not confined to rural areas. LISA analysis of *R*_*D*_ (Figure 7a) reveals more numerous clusters of high values than for *R*_*A*_, located in cities and larger towns. The spatial overlap with the destination-density map (Figure 6a) is striking: high bus-mediated accessibility coincides with high destination density. Two non-exclusive mechanisms can produce this pattern: bus operators may design routes to serve clusters of destinations such as hospitals and schools, and conversely destinations may be sited where bus services already exist because that is also where population density or footfall is high. The London cluster is again the largest, but is smaller in extent than its *R*_*A*_ counterpart, and is bordered by an extensive low-value cluster covering much of the suburbs, suggesting a relatively rapid drop-off in bus-service quality away from city centers. A similar pattern emerges for healthcare alone (Figure 7b), with the main qualitative difference a greater incidence of high–low outliers, possibly reflecting the fact that newer hospitals are often located on the edges of larger towns. Across all categories the distribution of *R*_*D*_ is similar (Figure 8), particularly in rural areas. Distributions for urban areas are roughly four times wider than those for rural areas and have a higher median; however, they are also strongly right-skewed, so better-than-average urban performance is concentrated in a small number of locations rather than uniformly distributed. Healthcare is the most unequal category, combining the widest spread with the highest peaks; education has the lowest and most compressed interquartile range, consistent with primary schools typically lying within walking distance of residential areas.

**Figure 6:**
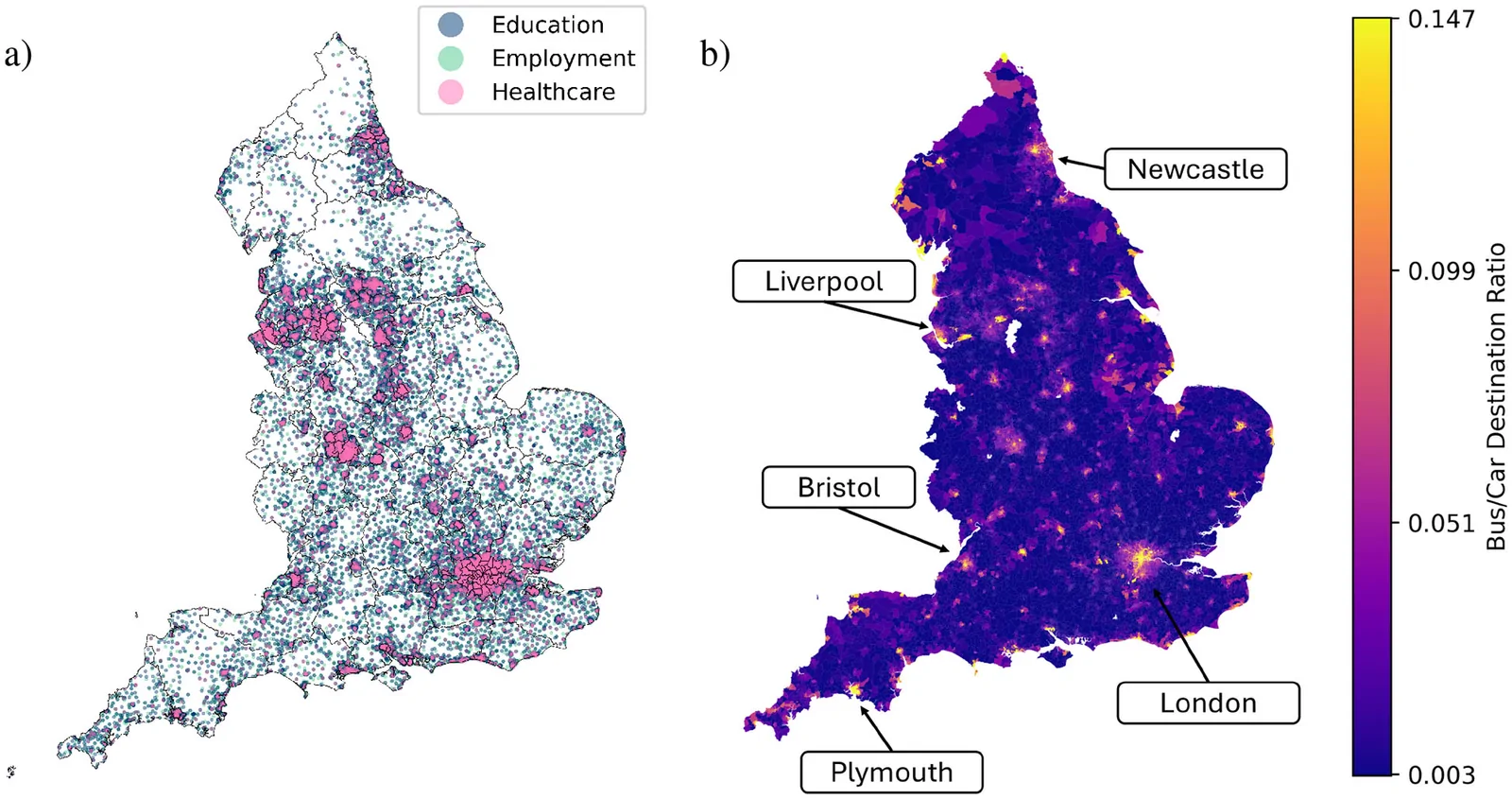
Destination accessibility maps. Although the area accessible within 40 min of travel is important, having access to a larger area does not necessarily imply being able to access more destinations. **(a)** Here, we study the number of destinations contained within each 40-min isochrones across education, employment and healthcare. **(b)** We calculate the ratio *R*_*D*_ of destinations that are contained within a 40-min bus isochrone divided by the number of destinations within a 40-min car isochrone. Large metropolitan areas exhibit high values, but we also observe that smaller urban areas achieve comparatively large values of *R*_*D*_. Note: the color scale is clipped between the 5th and 95th percentiles of *R*_*D*_. LSOAs with values outside this range are shown in the endpoint colors.

**Figure 7:**
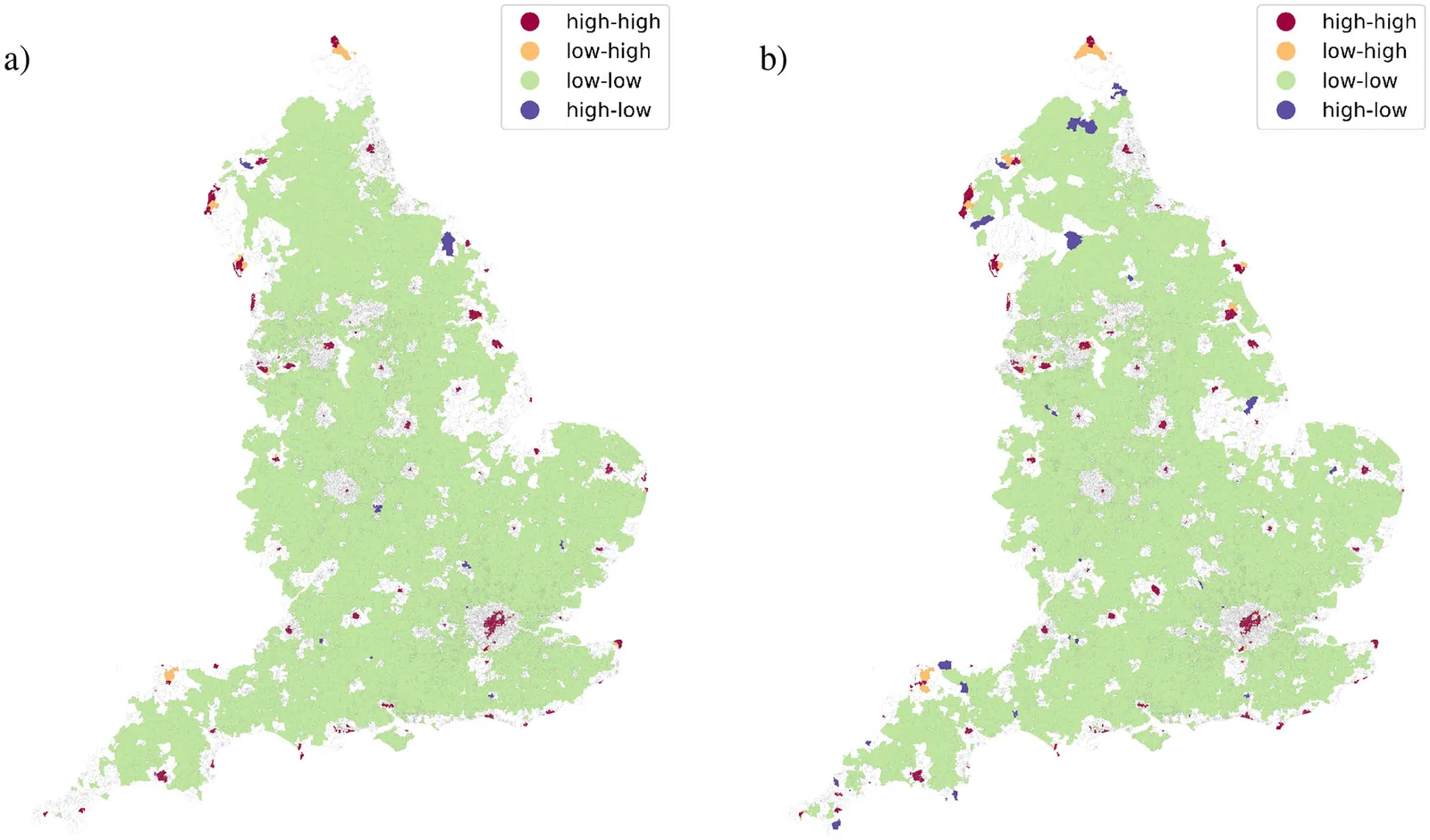
Destinations accessibility clustering. We depict the spatial clustering of the overall *R*_*D*_ ratio **(a)**, as well as when considering only healthcare destinations **(b)**. We observe a number of clusters with high *R*_*D*_ values across both maps. Maps for employment and education destinations are shown in the Supplementary material.

**Figure 8:**
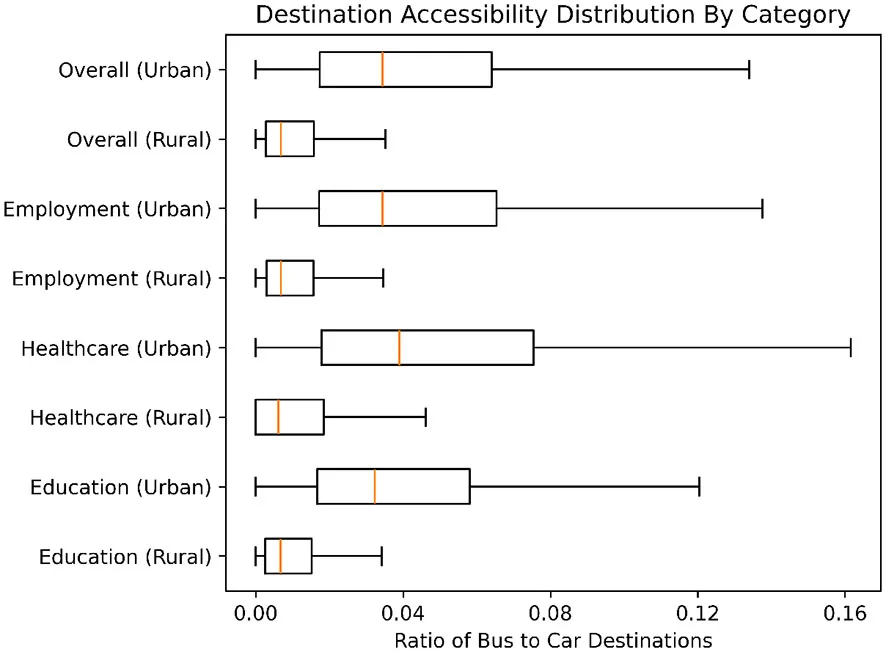
Urban and rural destination accessibility. Urban LSOAs exhibit much broader distributions of *R*_*D*_, achieving much larger values compared to rural LSOAs. Across all categories they also achieve a larger median value, indicating the better accessibility by bus that can be achieved in some (though not all) urban areas. Note that individual outlier points beyond 1.5 times the interquartile range are hidden for visual clarity. The boxes, medians and whiskers are computed on the full sample.

### 3.3 Comparing the two indicators

A high *R*_*A*_ does not imply a high *R*_*D*_. Figure 9a shows the joint distribution: for *R*_*A*_ > 0.05 there is a clear positive relationship, but the highest *R*_*D*_ values occur at much lower *R*_*A*_, pointing to geographic compactness: frequent, well-connected services concentrated in a small area as the key enabler of bus-mediated destination access, most likely in city centers. Beyond this regime, the relationship between *R*_*D*_ and *R*_*A*_ appears to flatten: at higher values of *R*_*A*_, the highest observed *R*_*D*_ do not increase anymore. This appears consistent with the hypothesis that enlarging the bus-reachable area adds progressively fewer additional destinations than improving frequency would. By settlement type, rural LSOAs cluster tightly near the origin where both ratios are low, but the area–destination relationship is itself stronger in rural areas (Pearson *r* = 0.59, *p* < 0.001) than in urban ones (Pearson *r* = 0.44, *p* < 0.001), implying that in rural settings expanding coverage and expanding destination access are more tightly coupled. When we compare bus-isochrone size directly to the number of reached destinations (Figure 9b), the positive linear relationship is much stronger (Pearson *r* = 0.85, *p* < 0.001), and is driven primarily by urban LSOAs that lie above the line of best fit. Rural LSOAs rarely exceed 100 reached destinations and underperform the line as the isochrone grows, indicating that rural bus services tend to serve a small number of destinations along long routes. The corresponding car relationship is strikingly weak (Pearson *r* = 0.17, *p* < 0.001): car accessibility is uniformly high regardless of location, while bus accessibility is sensitive to network structure; we could therefore hypothesize that improving bus-network design, not expanding road infrastructure, may be the more effective lever for closing the bus–car accessibility gap. However, testing this directly is beyond the scope of this study.

**Figure 9:**
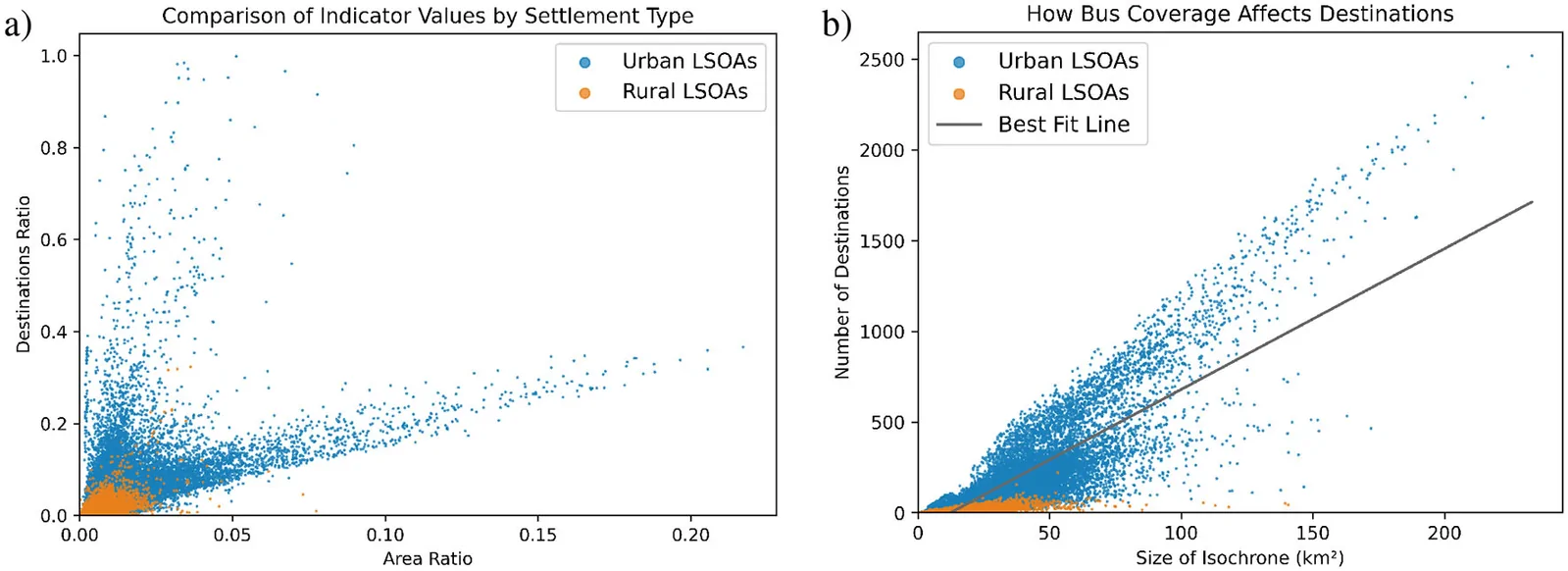
Area and destination accessibility comparison. **(a)** We analyse the relationship between the area of the isochrones and the number of destinations contained within them across rural and urban LSOAs separately. For visual clarity, the axes are truncated at *R*_*A*_ = 0.3 and *R*_*D*_ = 1; the 41 LSOAs with *R*_*D*_ > 1 (all in central-London boroughs such as Westminster, Waltham Forest and Tower Hamlets, where the bus network reaches more destinations than driving presumably due to the road network and restrictions on driving) and the 2 LSOAs with *R*_*A*_ > 0.3 are visible in the full plots in the Supplementary material. We note that rural LSOAs cluster near the origin, whereas urban LSOAs achieve higher values across both ratios. **(b)** The number of destinations within an isochrone increases as the isochrone gets larger, primarily in urban LSOAs.

### 3.4 Regional inequalities

Although national policy is set centrally, service planning and most subsidies sit with local transport authorities (Reardon and Benson, 2025). We therefore aggregate *R*_*D*_ to the regional and county/unitary-authority level (Figure 10). London has the highest regional mean *R*_*D*_(0.095), roughly 1.7 times the next-highest regional mean (South West, 0.057) and around 2.4 times the median of regional means. Counterintuitively, it also exhibits the largest internal inequality: it has the highest standard deviation of any region (0.165) and a substantial mean–median gap (mean 0.095 vs. median 0.058). The regional mean is pulled up by a small number of central boroughs (e.g. Westminster (0.378), Tower Hamlets (0.362) and Waltham Forest (0.238)), and 8 of the 10 best-performing areas in England by mean *R*_*D*_ are within London, while the lower median places most of London close to the national mean of 0.048.

**Figure 10:**
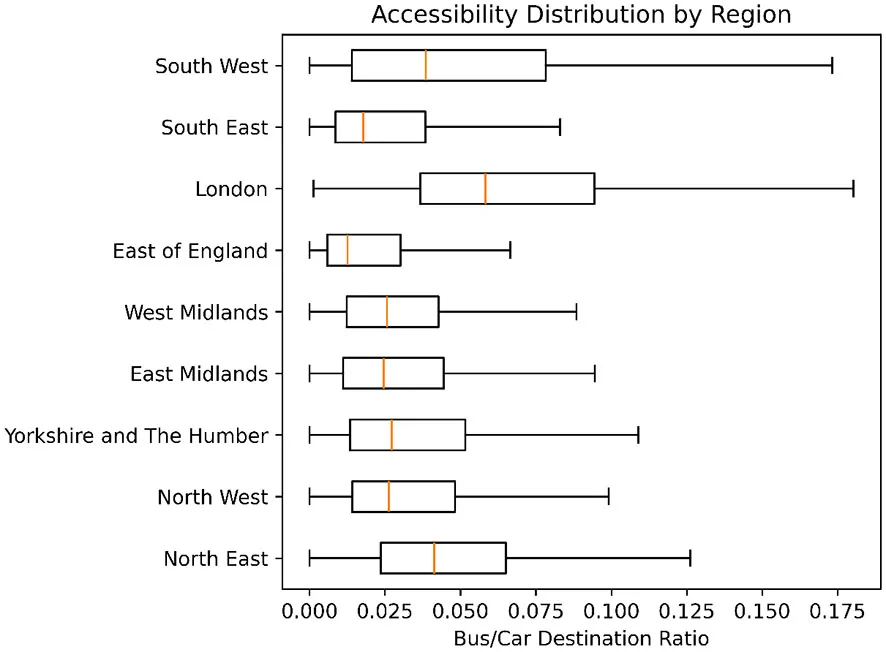
Regional accessibility. Local transport authorities often operate at the regional and local authority level. Thus, we show here the accessibility distribution of *R*_*D*_ by region. London exhibits the largest mean and median values compared to other regions. It also has the largest standard deviation of all regions. Interestingly, the South West also achieves large values, despite being a mainly rural region, thanks to a large *R*_*D*_ in the city of Plymouth. Note that individual outlier points beyond 1.5 times the interquartile range are hidden for visual clarity. The boxes, medians and whiskers are computed on the full sample.

The South West is also a notable outlier (Figure 10), despite being predominantly rural. Its average is driven mainly by Plymouth, with an average *R*_*D*_ of 0.199 and a median of 0.206, which is the highest of any English city outside London and reflects substantial recent investment in city bus services (Robins and Starr, 2025). Surrounding rural Devon and Cornwall, by contrast, score close to the regional median (0.034 and 0.043). The popular “North vs. South” framing is less informative in our data than proximity to a major city: the worst-performing counties are predominantly commuter regions in the South (Hertfordshire (0.008), Hampshire (0.013), Worcestershire (0.013), Warwickshire (0.014)), where historical underinvestment in bus services has produced large accessibility gaps. Rural counties more generally have lost an estimated 50–60% of their bus services between 2010 and 2023, with only a partial post-COVID recovery (Friends of the Earth, 2023). Within-region variation can also be substantial: in the North East, Middlesbrough scores 0.065 while Stockton-on-Tees and Redcar and Cleveland score 0.029 and 0.037 despite sitting under the same transport authority, suggesting that within-authority variation of this magnitude warrants further empirical investigation into whether it reflects local policy differences or unobserved compositional variation.

### 3.5 Determinants of accessibility disparities

The regional patterns of Section 3.4 describe *where* accessibility disparities occur, but not how they are distributed across areas with different socioeconomic characteristics. A long-standing question in the transport-poverty literature is whether transport-based measures of accessibility capture a dimension of disadvantage that is already reflected in conventional socioeconomic deprivation indices, or whether they measure something distinct (Lucas et al., 2012; Martens, 2016). We test this directly by combining *R*_*D*_ with area-level covariates drawn from the 2025 English Index of Multiple Deprivation (IMD) (Ministry of Housing, Communities and Local Government, 2025), which aggregates income, employment, education, health, crime, housing, and living-environment sub-domains at LSOA level, and from the 2021 UK Census. Across all English LSOAs, *R*_*D*_ is only weakly correlated with the overall IMD score (Pearson *r* = 0.14, *p* < 0.001; *r* = 0.18 in rural and *r* = 0.11 in urban LSOAs). Higher IMD values denote higher deprivation, so the positive sign implies that more-deprived LSOAs tend to have marginally *better*, not worse, bus-mediated accessibility. This pattern is consistent with the fact that deprived urban areas in England tend to be inner-city with dense bus networks. Figure 11 shows the joint distribution: urban LSOAs occupy the full IMD range at *R*_*D*_ values that span more than three orders of magnitude, while rural LSOAs cluster at lower *R*_*D*_ (< 10^−2^ for most) across a narrower deprivation range. The bivariate association is small in magnitude, so *R*_*D*_ and IMD carry largely independent information, consistent with *R*_*D*_ capturing a dimension of area-level disadvantage not reflected in conventional deprivation scores.

**Figure 11:**
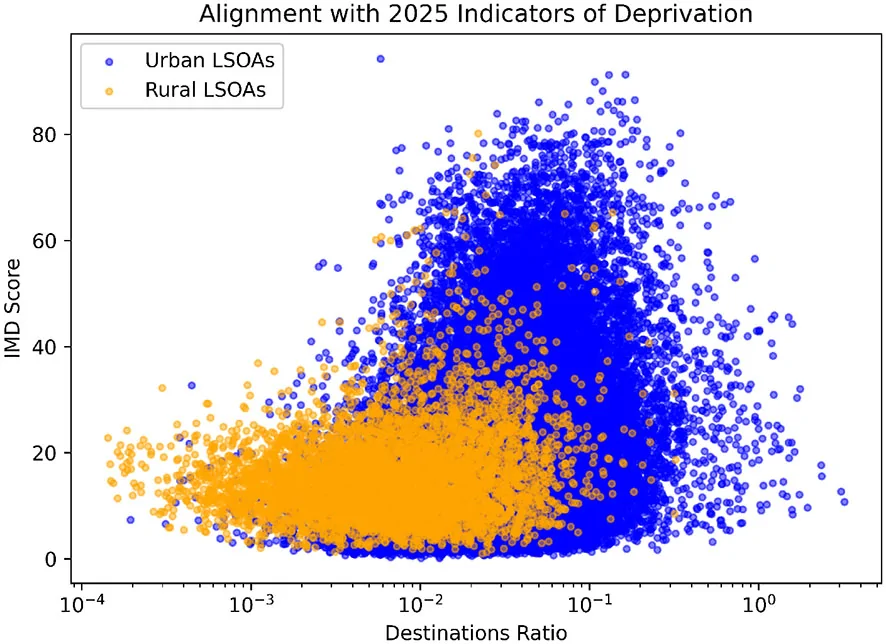
Destination-count ratio *R*_*D*_ (log scale) against the 2025 English Index of Multiple Deprivation score, for all English LSOAs. Points are colored by settlement type; higher IMD values indicate greater deprivation. LSOAs at any given IMD level span more than three orders of magnitude in *R*_*D*_.

#### 3.5.1 Multivariate model of *R*_*D*_

To move beyond bivariate description, we regress log(*R*_*D*_) on settlement type (a rural indicator derived from RUC21), the IMD score, the LSOA-level share of households without access to a car or van (from Census 2021 table TS045), and population density derived from the Census 2021 population and the LSOA polygon area. We include an interaction between IMD and the rural indicator to test whether the deprivation–accessibility association differs between settlement types, and z-standardize all continuous predictors so that coefficients can be compared directly. Lagrange-multiplier diagnostics on the ordinary-least-squares residuals (Table 1) reject the null of no spatial dependence in *both* the residual structure (robust LM-Error = 4, 446, *p* < 0.001) and the outcome itself (robust LM-Lag = 942, *p* < 0.001). Following Anselin (1988) and Elhorst (2010), this pattern motivates a combined spatial-autoregressive-with-error (SARAR) specification as the primary model, as described in Section 2.4.

Table 2 reports all four specifications side-by-side. Under the preferred SARAR specification, four findings are robust across all four models. First, rural LSOAs have substantially lower *R*_*D*_ than urban LSOAs: the SARAR coefficient is β_rural_ = −0.69 (*p* < 0.001), implying that a rural LSOA has roughly half the destination-count ratio of an otherwise-comparable urban LSOA. Settlement type is the single largest driver of the accessibility disparity. Second, the LSOA-level share of car-less households is the strongest positive predictor of *R*_*D*_: a one-standard-deviation increase (~16 percentage points) is associated with a ~46% direct increase in *R*_*D*_ under SARAR (β = +0.38, *p* < 0.001). This alignment is not an artifact of density or deprivation (both controlled in the model): it is specifically indexed by transport-related need, though the analysis cannot distinguish whether the association reflects demand-responsive service provision, residential sorting of car-less households toward better-served areas, or both. Third, IMD has a small negative main effect on *R*_*D*_ (β = −0.13 under SARAR, ~13% reduction per standard deviation of deprivation): once car-less-household share is held constant, more deprived LSOAs are marginally less, not more, accessible by bus, the opposite sign to the bivariate correlation, and a clear example of confounding between deprivation and car ownership. Fourth, population density is not a significant predictor once car-less-household share is included, in any specification: it is the composition of households, not raw density, that predicts bus accessibility. Both spatial parameters in the SARAR are highly significant and substantively meaningful. The spatial-lag parameter ρ = 0.42 (*p* < 0.001) indicates that an LSOA's *R*_*D*_ varies strongly with the average *R*_*D*_ of its neighbors, consistent with buses serving clusters of adjacent LSOAs together rather than each in isolation. The spatial-error parameter λ = 0.54 (*p* < 0.001) implies that even after conditioning on the covariates and the outcome lag, the residual signal remains strongly spatially clustered, consistent with substantial unmeasured local determinants of *R*_*D*_ such as operator geography, historical route inheritance, and local regimes. Both are considerably attenuated relative to their values in the single-parameter models (SEM λ = 0.77; SLM ρ = 0.50), confirming that when both are estimated jointly each absorbs part of what the other otherwise captured on its own. The IMD × rural interaction is the one estimate that varies across specifications. Under OLS the interaction is positive and substantively large (β = +0.28, *p* < 0.001), implying that the deprivation gradient is much steeper in rural than urban LSOAs; the SEM finds no interaction; the SLM finds a moderate positive interaction; the preferred SARAR resolves the ambiguity by returning a small but statistically significant positive effect (β = +0.06, *p* = 0.007). Under SARAR, the effective IMD slope is −0.13 in urban LSOAs and −0.13+0.06 = −0.07 in rural LSOAs: negative and significant in both, but modestly attenuated in rural areas. The mechanism producing this modest attenuation cannot be identified from the present cross-sectional analysis; possibilities include historical targeting of public subsidy toward rural transit provision, or compositional differences between deprived rural and deprived urban LSOAs that the RUC dichotomy does not capture.

#### 3.5.2 Decomposition into bus- and car-side effects

Because *R*_*D*_ is a ratio of bus-reachable to car-reachable destinations, any positive coefficient on *R*_*D*_ could in principle reflect either an increase in bus-reachable destinations (numerator) or a decrease in car-reachable destinations (denominator), or both. This ambiguity is particularly acute for the car-less-household coefficient: the LSOAs with the highest car-less-household shares are almost all central-urban, and Figure 3 shows that these are also the LSOAs where car isochrones may be compressed by low speed limits, restricted access and one-way street layouts. The positive *R*_*D*_–carless association could therefore reflect either bus operators responding to demand, or the same central-urban land-use structure constraining car access, or a combination. To distinguish these mechanisms, we refit the same spatial-error specification with log(total bus destinations+1) and log(total car destinations+1) as separate outcomes. Results in Table 3 are based on the SEM specification because SEM does not include a spatial lag of the outcome, so the difference-of-coefficients identity $\beta^{R_{D}}\approx \beta^{\text{bus}}-\beta^{\text{car}}$ holds directly at the level of the estimated coefficients (under SARAR the identity would hold only after computing total effects for each fit). The four-way estimates for the ratio itself remain those in Table 2.

The results from the decomposition (Table 3) help better understand our overall results in a number of ways. First, the car-less-household coefficient is almost entirely a bus-side effect: a one-standard-deviation increase in car-less-household share is associated with a ~69% increase in the number of bus-reachable destinations (β_bus_ = +0.52), against a much smaller ~4% increase in car-reachable destinations (β_car_ = +0.04). The positive *R*_*D*_-carless-share association is therefore predominantly located on the bus side of the ratio rather than reflecting compressed car reach in the same LSOAs. Second, the rural penalty is also concentrated on the bus side (β_bus_ = −0.98 vs. β_car_ = −0.06): rural LSOAs reach approximately the same number of car destinations as urban LSOAs on average, but reach around two-thirds fewer bus destinations. Third, the IMD main effect is likewise a bus-side phenomenon (β_bus_ = −0.22 vs. β_car_ = −0.02): area-level deprivation, controlling for car-less-household share and rural status, is specifically associated with reduced bus-network coverage of destinations, and not with any change in car reach.

As a completeness check, we refit the same specification with log(*R*_*A*_) as outcome (Supplementary Table 5). The covariate profile is broadly similar to that of *R*_*D*_, with one notable exception: the rural coefficient under SARAR is more than an order of magnitude smaller for *R*_*A*_ than for *R*_*D*_ (β = −0.04 versus β = −0.69; both *p* < 0.001). This asymmetry mirrors the bus-side decomposition in Table 3: the rural disadvantage in bus-mediated accessibility manifests almost entirely through the number of destinations reached, rather than through the geographic extent of the bus-reachable area.

Taken together, the SARAR multivariate results and the SEM-based decomposition give us a coherent picture. Bus-mediated accessibility in England is systematically higher where car-less households are more prevalent, driven by the geography of the bus network itself rather than by ratio-level artifacts of car access. The rural–urban and deprivation–accessibility gaps documented in Sections 3.1–3.4 are properties of the bus network specifically, and cannot be closed by intervention in the road network alone. Both spatial parameters provide relevant information: ρ = 0.42 indicates that an LSOA's *R*_*D*_ is spatially correlated with the average *R*_*D*_ of its neighbors (under queen-contiguity); λ = 0.54 indicates that residual unobserved spatial structure persist even after conditioning on the covariates and the outcome lag.

## 4 Discussion

Bus-mediated transport disadvantage in England is widespread rather than localized. Even with a generous 40-minute travel-time budget, a large majority of LSOAs reach only a small fraction of the destinations accessible by car within the same window (Section 3), and clusters of low *R*_*A*_ and *R*_*D*_ span most of rural England rather than being confined to specific regions or settlement types. Because we obtain these results at LSOA resolution across the whole of England, rather than in a single case-study city, they provide direct evidence at national scale for the widespread transport disadvantage previously documented in the UK transport-poverty literature (Gates et al., 2019) and in international work on accessibility justice and transport equity (Lucas et al., 2012; Martens, 2016). The national spread of the disadvantage is consistent even after adjustment for settlement type, deprivation, car-less-household share and population density in the multivariate regression of Section 3.5.1. Beyond confirming the geographical spread, our analysis of the covariates of *R*_*D*_ produces two findings that help us understand how bus-mediated accessibility should be interpreted as a dimension of area-level disadvantage. First, the LSOA-level share of car-less households is the strongest positive predictor of *R*_*D*_: bus-mediated accessibility is systematically higher where a larger share of the local population most directly depends on it, an association that is neither an artifact of density (which is non-significant once car-less-household share is included) nor of general deprivation (which enters the model with a small negative coefficient). This alignment is consistent with either demand-responsive service provision or residential sorting of car-less households toward better-served areas; our cross-sectional analysis cannot distinguish between these mechanisms. Second, *R*_*D*_ is only weakly correlated with the Index of Multiple Deprivation (*r* = 0.14 nationally at the bivariate level), and the multivariate regression retains only a small residual IMD effect once car-less-household share and settlement type are controlled. Taken together, these results provide national-scale empirical support for the view that mobility-mediated accessibility constitutes a distinct dimension of area-level disadvantage that conventional income- and education-weighted deprivation indices do not fully capture. A further decomposition of the ratio into its bus-side and car-side components (Section 3.5.2) shows that the rural, IMD, and car-less-household effects on *R*_*D*_ all operate almost exclusively on the bus side: the disparity is a property of the bus network specifically, not of the road network. These findings suggest several policy hypotheses, which we present as such rather than as established causal claims. The weak correspondence between *R*_*A*_ and *R*_*D*_, the apparent flattening of *R*_*D*_ at higher *R*_*A*_ values, and the very weak relationship between car-isochrone area and reached destinations, are together consistent with the idea that service frequency, route design and proximity to destination clusters may be more consequential levers for bus-mediated accessibility than simply expanding geographic coverage. At regional level, *R*_*D*_ varies by orders of magnitude across regulatory regimes: London, which operates a franchised bus network under Transport for London rather than the deregulated arrangements that apply elsewhere in England, exhibits *R*_*D*_ values an order of magnitude higher than most of the country. This scale of difference suggests that regulatory arrangements, in addition to local investment decisions, plausibly play a substantial role in shaping bus-mediated accessibility. At sub-regional scale, substantial variation in *R*_*D*_ can be observed between authorities that share the same regional transport regime; Plymouth, for example, is the highest-scoring English city outside London on *R*_*D*_ and coincides with a documented period of local bus-partnership investment (Robins and Starr, 2025), though our cross-sectional analysis cannot establish whether the investment produced the accessibility outcome, whether both reflect a shared underlying factor, or the direction of any causal relation. Follow-up work with panel data across intervention timings would be required to make causal claims of this kind. Our findings are subject to a number of limitations. First, the analysis is restricted to bus services and excludes metro and rail; in cities with substantial alternative public-transport modes (e.g. London and Manchester) actual accessibility for populations without a car will be higher than *R*_*A*_ and *R*_*D*_ suggest. The focus on buses is deliberate, reflecting the fact that buses are the only public-transport mode with near-universal national coverage and that the populations most exposed to transport poverty disproportionately rely on them (Gates et al., 2019). Second, our routing engine models car travel under free-flow speeds and is invariant to departure time; in dense urban centers at peak times this will bias *R*_*A*_ and *R*_*D*_ downwards (i.e., understate the relative performance of buses) relative to what real peak-hour car access would deliver, and comparisons across regulatory regimes should be read with this asymmetry in mind. Third, the analysis is cross-sectional and treats scheduled bus timetables as ground truth, whereas real-world deviations from schedule are known to exist and may vary systematically across the urban–rural divide (Chen and Botta, 2025, 2026); integrating GPS-corrected timetables would refine absolute values but the general patterns reported here are unlikely to be overturned. Fourth, *R*_*D*_ measures the coverage of destination *points* rather than the volume of jobs, pupils or patients that each destination represents. JTS 2019 provides employment centers in three ordinal size bands but does not report continuous job counts or per-establishment capacity for schools, GPs and hospitals; a five-scheme weighting sensitivity (Supplementary Table 6) shows the count-based employment ratio is robust to alternative weight vectors spanning a 1:1 to 50:1 large-to-small range, but a fully capacity-weighted measure would additionally require linking external datasets such as the Get Information About Schools register for pupil numbers and NHS Digital list-size / bed-capacity statistics for healthcare, which we leave as a natural extension. Fifth, the destination dataset is drawn from the 2019 edition of the Journey Time Statistics while the transport-network inputs reflect a January 2026 snapshot; changes in the underlying geography of schools, healthcare provision and employment centers over that interval are not captured, though they are unlikely to reverse the qualitative patterns reported here. As with any spatial analysis carried out at a single geographic granularity, the results are potentially subject to the modifiable areal unit problem: the same underlying data aggregated at a different level (e.g. output areas, MSOAs) would produce numerically different indicator values and different covariate coefficients. In addition, we use a single population-weighted centroid per LSOA as the origin for routing, which is an accurate approximation for compact urban LSOAs but a coarser one for the largest rural LSOAs, where residents may live several kilometers from the centroid and therefore have even lower bus reach than the centroid measurement suggests. This could be partially addressed by replicating this analysis at finer geographic units (output areas, 100 meter grid cells). Sixth, the cross-sectional design precludes causal identification of the mechanisms behind our covariate associations, and the IMD × rural interaction is specification-sensitive across the spatial models we compare (Section 3.5.1). Finally, ~0.3% of LSOAs produced degenerate or implausible isochrones and were excluded; sensitivity analyses indicate that snapping origin centroids to the road network would mitigate this, and we recommend this step for future implementations. The methodology developed here suggests several extensions. First, broadening the temporal coverage (off-peak, evening, weekend, and bank-holiday services) would highlight accessibility differences across the working week that impact different segments of the population differently, from night-shift workers to caregivers with variable schedules. Using empirically-derived rather than scheduled travel times, drawing on recent techniques for GPS-corrected timetables (Chen and Botta, 2026), would refine the levels of accessibility measured. Second, given larger compute resources, the methodology applies straightforwardly to finer geographic units (census output areas, 100 m grid cells), surfacing the intra-suburb heterogeneity that our LSOA-level analysis can only hint at. Third, combining *R*_*A*_ and *R*_*D*_ with fare and detailed population data would tighten the social-impact framing further, for instance by weighting accessibility by the population at risk of transport poverty rather than by LSOAs. Finally, allowing the covariate effects identified in Section 3.5.1 to vary in space using geographically weighted regression, or extending to a spatial-panel design that exploits within-authority variation over time, would help identify whether the intra-authority differences we observe reflect local policy choices or unobserved compositional differences. All analysis code and processed LSOA-level indicators are available in the public repository (Townley, 2026), to support replication and adaptation of the approach to other geographic settings.

## 5 Conclusion

We have presented a national-scale analysis of bus-mediated accessibility in England, combining a pair of complementary isochrone-based indicators with a multivariate spatial regression at LSOA granularity. The analysis surfaces patterns that case-study designs are unable to capture: bus-mediated transport disadvantage is widespread rather than localized, and disproportionately affects rural populations. At the LSOA level, the share of car-less households is the strongest positive predictor of the destination-count ratio, and a decomposition of the ratio into its bus and car components shows that the rural, deprivation and car-ownership effects all operate on the bus side, indicating that the disparity is a property of the bus network rather than of the road network. Because the destination-count ratio is only weakly correlated with the Index of Multiple Deprivation, our results provide national-scale empirical support for the view that mobility-mediated accessibility constitutes a distinct dimension of area-level disadvantage that conventional deprivation indices do not fully capture. Methodologically, the pipeline is open, scalable and transferable (Townley, 2026), and forms a step toward mobility-aware indicators of social impact at policy-relevant geographic granularity.

## Data Availability

The original contributions presented in the study are included in the article/Supplementary material, further inquiries can be directed to the corresponding author.
