# Supplementary material for: How transport coverage shapes access to opportunities: a national-scale isochrone analysis of bus network accessibility in England
Source: Front Big Data. 2026 Oct 22;9:1942887. doi: 10.3389/fdata.2026.1942887 (PMC13735104; doi:10.3389/fdata.2026.1942887)
Supplement: Supplementary file 1 [file Supplementary_file_1.pdf]

SUPPLEMENTARY FIGURES

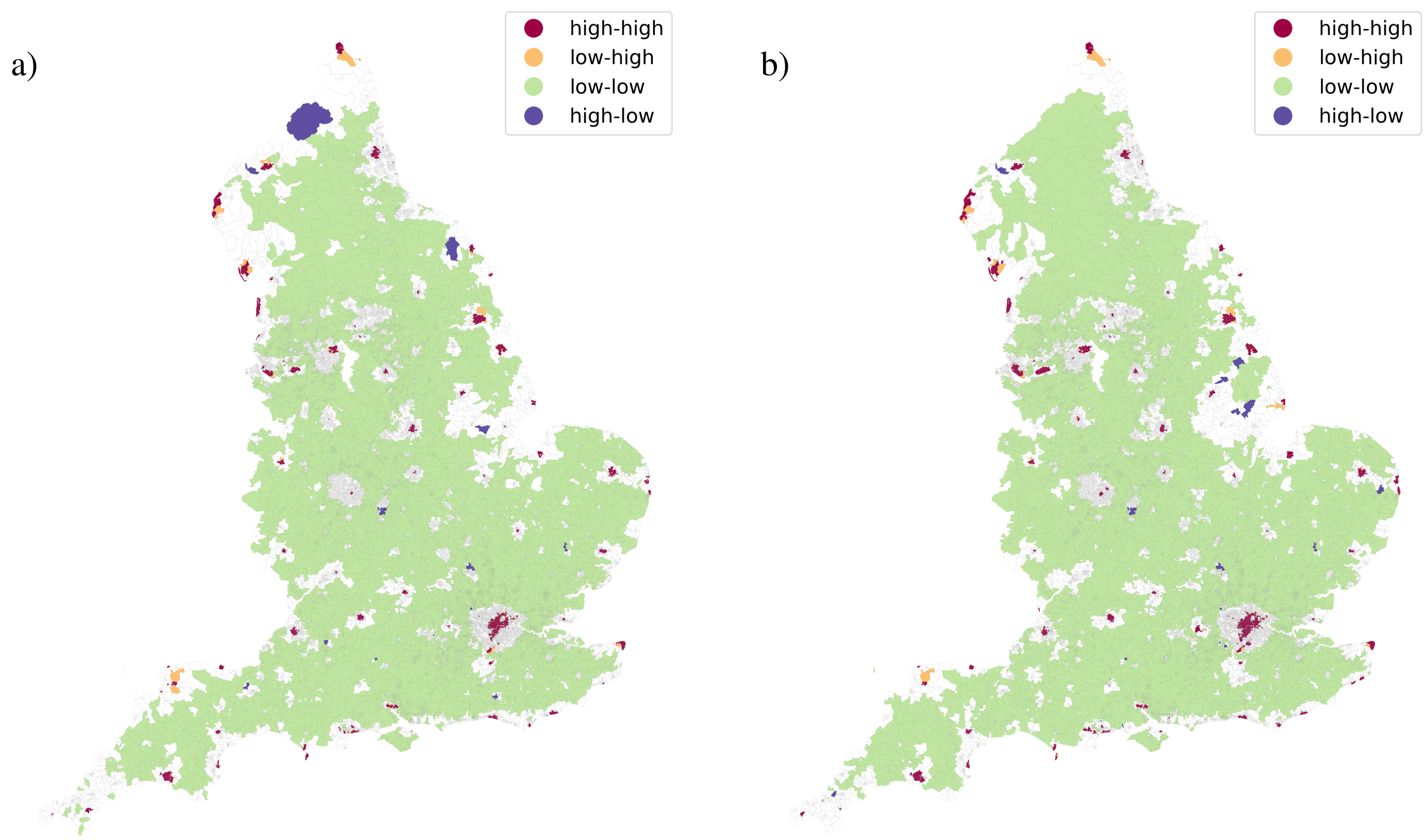

Figure 12. Local clustering of destination ratios across England, complementing Figure 7: (a) employment ratio, (b) education ratio.

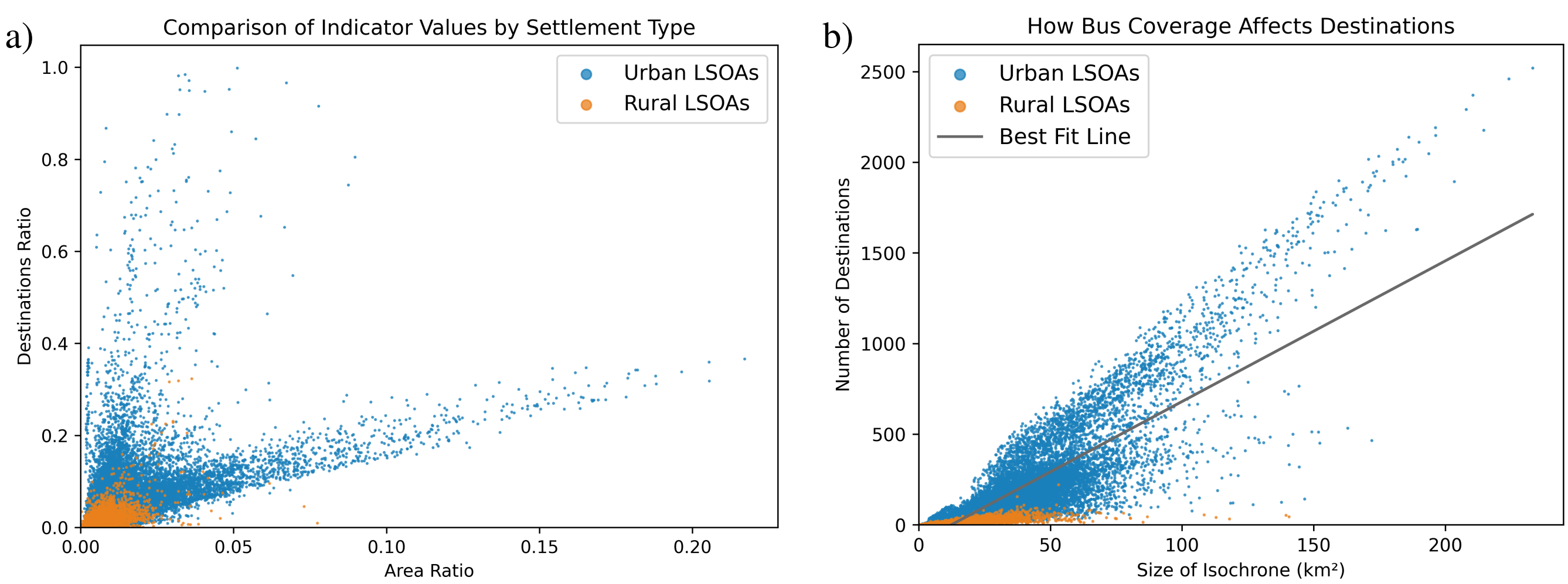

Figure 13. Full versions of the scatterplots in Figure 9, with no outliers removed: (a) accessibility-indicator pair, (b) bus-isochrone area against number of reached destinations.

Table 4. Robustness of the SARAR estimates in Table 2 to the choice of spatial weights matrix. All models are fitted with the same specification (rural, IMD, car-less-household share, population density, and IMD × rural interaction; continuous predictors z-standardised) and the same estimator (`spreg.GM_Combo_Het`) but different row-standardised weights: queen contiguity (baseline), $k = 4$ nearest neighbours by population-weighted-centroid distance, and $k = 8$ nearest neighbours. Signs, statistical significance, and relative ordering of the covariate effects are preserved across all three specifications. The SARAR spatial-lag parameter ρ is essentially constant across weights choices; the spatial-error parameter λ increases with neighbourhood density, reflecting that a more permissive definition of neighbours attributes a larger share of residual variance to spatial clustering. Standard errors in parentheses. N = 33,657.

	Queen		KNN, $k = 4$		KNN, $k = 8$	
Predictor	β	SE	β	SE	β	SE
Rural	-0.693	(0.026)	-0.863	(0.029)	-0.867	(0.028)
IMD (z, urban)	-0.134	(0.005)	-0.162	(0.006)	-0.188	(0.006)
Car-less household share (z)	+0.380	(0.009)	+0.428	(0.009)	+0.462	(0.009)
Population density (z)	+0.001	(0.002)	+0.005	(0.003)	+0.008	(0.004)
IMD × Rural	+0.064	(0.024)	+0.072	(0.024)	+0.069	(0.024)
ρ (spatial lag of y)	+0.424	(0.013)	+0.432	(0.014)	+0.402	(0.014)
λ (spatial error autocorrelation)	+0.540	(0.014)	+0.653	(0.013)	+0.799	(0.012)

Table 5. Supplementary regression: SARAR estimates for $\log R_A$ compared with the SARAR estimates for $\log R_D$ (reproduced from Table 2). Specification, covariates, sample and weights are identical to those used in the main regression. GMM standard errors in parentheses. Full OLS/SEM/SLM results for $\log R_A$ are available in the code repository (Townley, 2026). N = 33,657.

	$\log R_A$		$\log R_D$	
Predictor	β	SE	β	SE
Intercept	-1.934	(0.045)	-1.965	(0.047)
Rural	-0.042	(0.012)	-0.692	(0.026)
IMD (z, urban)	-0.109	(0.004)	-0.134	(0.005)
Car-less household share (z)	+0.292	(0.006)	+0.380	(0.009)
Population density (z)	-0.002	(0.002)	+0.001	(0.002)
IMD x Rural	+0.069	(0.014)	+0.064	(0.023)
ρ (spatial lag of y)	+0.594	(0.009)	+0.424	(0.013)
λ (spatial error autocorr.)	+0.335	(0.015)	+0.540	(0.014)

Table 6. Sensitivity of the employment-specific destination-count ratio to alternative job-count weighting schemes. The Journey Time Statistics 2019 employment dataset provides only ordinal size bands (small: 100–499 jobs; medium: 500–4,999; large: $\geq 5{,}000$) rather than per-centre job counts. We evaluate four weighted variants spanning a 1:1 to 50:1 large-to-small weight ratio alongside the count-based baseline used in the main paper. Weight vectors are (small/medium/large) jobs. Correlations are with the count-based baseline over the full 33,658-LSOA sample.

Scheme	Weights (S/M/L)	Median	Mean	Q75	% <0.05	Pearson	Spearman
Count (baseline)	1/1/1	0.029	0.049	0.057	70.5	—	—
Lower-bound	100/500/5000	0.027	0.062	0.062	69.5	0.912	0.928
Midpoint	300/2750/7500	0.028	0.054	0.058	70.5	0.964	0.964
Upper-heavy	300/2750/15000	0.027	0.058	0.060	69.9	0.936	0.945
Log-midpoint	224/1580/8660	0.027	0.058	0.060	70.0	0.940	0.949

Table 7. Headline accessibility shares under LSOA weighting and Census 2021 population weighting. LSOAs in England are designed to have roughly equal resident populations (400–1,200 households each), so LSOA-share and population-share statistics are close in practice: the absolute difference is below one percentage point for every threshold reported in the main text.

Threshold	% of LSOAs	% of population	Difference (pp)
$R_A < 0.01$	61.4	60.9	-0.5
$R_A < 0.05$	97.8	97.8	-0.0
$R_D < 0.01$	19.6	19.6	+0.1
$R_D < 0.05$	71.0	70.6	-0.4
$R_D < 0.10$	89.6	89.4	-0.1

Table 8. Parameter-sensitivity check on the 483-LSOA sensitivity sample. Each scenario perturbs one dimension of the paper baseline (bus 25th percentile, car 50th percentile, 40-minute budget, Mon 08:30, bus 250 m / car 500 m grid). Median and 75th-percentile values of R_A and R_D are shown alongside the share of LSOAs below the headline thresholds ($R_A < 0.01$, $R_D < 0.05$) and Spearman rank correlation with the paper baseline on the same LSOAs. Values marked "baseline (restricted) report the paper baseline restricted to the 483 LSOAs.

Scenario	R_A			R_D			Spearman
	Median	Q75	% <0.01	Median	Q75	% <0.05	R_A/R_D
Baseline (restricted)	0.007	0.012	64.8	0.019	0.042	80.5	—
A Matched percentile (bus 50th)	0.005	0.008	81.6	0.014	0.030	85.3	0.947 / 0.919
B Short budget (20 min)	0.004	0.007	87.0	0.018	0.030	89.4	0.759 / 0.436
C Long budget (60 min)	0.015	0.024	31.1	0.036	0.093	58.8	0.888 / 0.864
D Off-peak (Mon 14:00)	0.007	0.012	66.9	0.020	0.041	81.4	0.976 / 0.926
E Weekend (Sat 10:00)	0.008	0.013	62.1	0.021	0.044	79.9	0.980 / 0.928
F Matched grid (250 m both)	0.007	0.012	65.8	0.020	0.041	81.2	0.999 / 0.941

Table 9. Global Moran's I for each principal accessibility indicator, computed on the same row-standardised queen-contiguity weights matrix as the multivariate regression (Isles of Scilly excluded, $N = 33{,}657$). Expected values under the null are all $-1/(N-1) \approx -3.0 \times 10^{-5}$; z-scores use the normal-approximation variance; and p-values are based on 999 random permutations of the outcome. All six indicators reject the null of no spatial autocorrelation at the smallest attainable permutation p-value.

Indicator	I	$\mathrm{E}[I]$	z	Permutation p
R_A	+0.836	-0.00003	+254.8	<0.001
R_D	+0.693	-0.00003	+211.3	<0.001
$\log(R_D + \varepsilon)$	+0.810	-0.00003	+246.9	<0.001
Employment ratio	+0.687	-0.00003	+209.6	<0.001
Education ratio	+0.707	-0.00003	+215.7	<0.001
Healthcare ratio	+0.645	-0.00003	+196.7	<0.001
